# “Metal Detector” Gene May Influence Lead Absorption: Variants Predict Higher Blood Lead Levels in Children

**Published:** 2008-09

**Authors:** Charles W. Schmidt

An estimated 310,000 U.S. children between ages 1 and 5 have elevated blood lead levels despite efforts to reduce lead in the environment. Research in the past decade has begun to focus on factors that could make some children more susceptible to lead poisoning even at low levels of exposure. A new study explores one such possible factor—gene variants that influence lead absorption—linking variants in two iron metabolism genes to higher blood lead levels in children **[*EHP* 116:1261–1266; Hopkins et al.]**.

When researchers analyzed umbilical cord blood from 422 children in Mexico, they found that the presence of two variants of the hemochromatosis (*HFE* ) gene—*HFE C282Y* and *HFE H63D*—predicted blood lead levels 11% higher than those in children not carrying the variants. Moreover, the presence of either *HFE* variant combined with a variant form of the transferrin (*TF* ) receptor gene—*TF-P570S*—predicted blood lead levels 50% higher than in children with none of the variants.

Although the *HFE* and *TF* genes normally regulate iron metabolism, they may also influence blood lead levels because lead—like iron—is a divalent metal. Thus, the two metals can be “mistaken” for each other during metabolic processes. The *HFE* gene regulates iron-binding proteins, including TF, and variant forms of this gene sometimes induce hemochromatosis, a disease characterized by increased intestinal absorption of iron that contributes to abnormally high iron stores in adulthood.

The authors hypothesized that the *HFE* variants might similarly increase absorption of lead, a hypothesis supported by the results of this study. *TF* interacts with *HFE* to form a complex that down-regulates iron absorption. However, *TF-P570S* may interact with the *HFE* variants in ways that heighten metal absorption rates. Study results showed the *TF* and *HFE* variants produced higher lead levels than those predicted by either *HFE* variant alone.

Previously published research by these investigators has shown that having the *HFE* variants predicted lower blood lead levels in elderly men compared with men without the variants. The contrasting findings, the authors speculate, may reflect age-specific differences in body iron stores and in the variants’ effect on lead metabolism. Among children with low iron body stores and high iron needs, the variants predicted higher blood lead levels. But as iron stores accumulate with age, the variants down-regulated iron and lead absorption, leading to progressive declines in blood lead levels. The study’s key implications are twofold: first, that children with variant iron-metabolizing genes may be especially susceptible to the effects of lead at low exposure levels, and second, that genetic variants may increase risk at one life stage and decrease it at others.

